# Regenerative Injection Treatments Utilizing Platelet Products and Prolotherapy for Cervical Spine Pain: A Functional Spinal Unit Approach

**DOI:** 10.7759/cureus.18608

**Published:** 2021-10-08

**Authors:** Christopher Williams, Mairin Jerome, Chaz Fausel, Ehren Dodson, Ian Stemper, Christopher Centeno

**Affiliations:** 1 Physical Medicine and Rehabilitation, Interventional Orthopedics and Regenerative Medicine, Interventional Orthopedics of Atlanta, Atlanta, USA; 2 Physical Medicine and Rehabilitation, Regenerative SportsCare Institute, New York, USA; 3 Physical Medicine and Rehabilitation, Advanced Regenerative Health, Denver, USA; 4 Research and Development, Regenexx, LLC, Des Moines, USA; 5 Physical Medicine and Rehabilitation, Centeno-Schultz Clinic, Denver, USA

**Keywords:** platelet-rich plasma (prp), opioids, opioid epidemic, prolotherapy, platelet lysate, orthobiologics, regenerative medicine therapies, interventional pain management, chronic pain, neck pain

## Abstract

Background

The increasing burden of musculoskeletal disorders combined with the high utilization of opiates and the relatively limited ability of traditional approaches to satisfactorily address many of these conditions has spurred an increased interest in alternative treatments such as regenerative medicine therapies. Evidence is growing to support the use of regenerative injection treatments, including prolotherapy, platelet-rich plasma (PRP), platelet lysate (PL), and mesenchymal stromal cells. This study aims to offer a proof of concept via a case series of patients with neck pain treated using a functional spinal unit (FSU) model with combination prolotherapy, PRP, and PL injections.

Methodology

A chart review identified patients with neck pain treated with a combination of cervical injections using concentrated platelets and prolotherapy.

Results

A total of 14 patients met the inclusion criteria. The average decrease in the Numeric Pain Score was 2.8 (p = 0.002). The mean decrease in the Functional Rating Index was 27.3 (p = 0.004) at 24 months. Two patients had mild adverse reactions.

Conclusions

This case series demonstrates basic safety and clinically significant improvements in patients treated for neck pain with autologous concentrated platelet products and prolotherapy utilizing an FSU treatment protocol. Additional clinical studies are warranted with a larger patient sample size and longer follow-up periods.

## Introduction

As people are living longer, musculoskeletal pathology has become the leading cause of disability worldwide, with back pain accountable as the leading driver of years lived with disability [1]. Though low back pain is the leading contributor to the number of years lived with disability, a review of global disease burden from 1990 to 2013 found neck pain to be the fourth most common cause of years lived with disability globally and second to low back pain for high-income countries [1]. The annual prevalence of neck pain is 13.7-50% with a mean lifetime neck pain prevalence of 48.5% [2-4]. Though episodic in nature with 33-65% of occurrences estimated to resolve within a year, relapses are common [4]. Neck and back pain have been linked to a 1.6-2.3-fold increase in mental health disorders such as depression, anxiety disorders, and substance abuse disorders [5]. Neck pain is also associated with the high utilization of opioid medications. Indeed, opiate use within the prior 30 days of 837 patients surveyed with chronic neck and/or back pain ranged from 34.5% to 52.4% [6].

Common causes of spine pain, both cervical and lumbar, include spondylosis, discogenic, facet-mediated, and myofascial pain [7,8]. The traditional treatment of neck pain refractory to conservative care with medication and physical therapy includes corticosteroid injections targeted to a limited number of structures, radiofrequency ablation, or in very severe or debilitating cases, surgery [9,10]. Facet joint injections, medial branch blocks, and radiofrequency ablation have become the mainstay of interventional pain management for axial neck pain with a utilization increase of 362.9% in the cervical spine from 2000 to 2014 in Medicare recipients[10]. This increase in utilization comes despite disappointing results when calculating cost and therapeutic efficacy, with evidence failing to demonstrate long-term relief from facet joint blocks and the need for repeated injections [10-13]. Recent studies also suggest that the corticosteroids and analgesics commonly utilized for these injections may contribute to the progression of arthritis [14-16]. While there is some evidence of longer-term relief from neurolysis techniques, it is possible that this could contribute to cervical deep segmental stabilizer denervation and atrophy as the cervical medial branches innervate the semispinalis capitis, semispinalis cervicis, and multifidus muscles [12,17]. Regarding surgery, only limited success has been demonstrated for the treatment of neck pain without severe neurological compromise, while conferring a much higher risk profile than percutaneous injections [18].

The increasing burden of musculoskeletal disorders combined with the relatively limited ability of traditional approaches, including treatment with opiates, to satisfactorily address painful conditions of the spine has spurred an increased interest in alternative treatments. One such category is regenerative injection treatment (RIT), defined as injections of proliferative solutions, growth factors, or cells intended to strengthen or repair injured or weak musculoskeletal and nervous structures [19]. Evidence is growing to support the use of RITs, including prolotherapy, autologous blood products such as platelet-rich plasma (PRP), whole blood, and platelet lysate (PL), in the treatment of musculoskeletal conditions [20-25]. These injectates have proposed mechanisms of action including the local introduction of supraphysiological concentrations of growth factors and bioactive proteins that induce activation of the immune system and the natural healing cascade [26-29].

The emergence of RITs has allowed for the traditional treatment philosophy for interventional pain management to expand beyond the “single pain generator” model to consider the entire osteoligamentous complex, known as the functional spinal unit (FSU) [30]. In such a model, multiple tissue types are considered and treated, including fascia, muscle, synovial joints, and ligaments. Here, we present a case series of patients with axial neck pain treated using an FSU model via percutaneous RITs, specifically a combination of prolotherapy, PRP, and PL, as an alternative to treatment with opioids and other traditional treatment methods.

## Materials and methods

Patient selection

Registry data collected at a single center were used to acquire outcomes data. Patients undergoing treatment at a private practice, outpatient-based interventional pain center were encouraged to enroll in a patient registry to track safety and outcomes data. All patients signed a written consent prior to participation, and the registry data protocol was approved by an Institutional Review Board (HHS OHRP #IRB00002637). The registry protocol included prospectively tracking patients’ outcomes using an electronic system, ClinCapture (Clinovo Clinical Data Solutions, Sunnyvale, CA), that automatically generates pre- and post-treatment questionnaires based on the body area treated. After treatment, patients received surveys to self-report complications and outcomes at one, three, six, twelve, eighteen, twenty-four months, and annually through 20 years. Up to five attempts were made to contact patients at each time point before being considered lost to follow-up. Adverse events were documented by asking patients about any complications after receiving the treatment to elicit potentially related issues, which were reviewed, adjudicated by the treating physician, characterized, and indexed.

Inclusion criteria for this study were as follows: patients with axial neck pain with or without radiculopathy treated via injections of PRP or PL to cervical facet joints with or without epidural overfill, cervical transforaminal epidural injections with PL, and cervical ligaments injections with combination prolotherapy with or without PL between August 2014 and March 2017. Only patients with available pre- and post-treatment outcome data to 24 months were included. Search criteria to identify patients within the registry included those with axial neck pain with or without radicular symptoms, cervical instability, foraminal or central stenosis, degenerative disc disease, and facet arthropathy, before a more extensive chart review was conducted confirming inclusion criteria.

Processing of the Platelet Products

In preparation for the treatment, patients were instructed to cease the use of non-steroidal anti-inflammatory drugs and corticosteroids for the preceding two weeks so as not to interfere with platelet activity. Within 24 hours of the treatment procedure, patients underwent a heparinized venous blood draw of approximately 60 mL. The PL and PRP were hand-processed in a sterile ISO-5-class biologic safety cabinet, the details of which have been previously described [23,31]. In brief, all blood products were processed by hand in a sterile ISO-5-class biologic safety cabinet. For PRP, whole blood was centrifuged at 200×g to separate the plasma and buffy coat layers from the red blood cells. The resultant supernatant was red cell and white cell-poor, with the volume dependent on individual patient hematocrit levels. To prepare the PL, the same process was followed to prepare the supernatant, which was then manually extracted under a sterile hood via a pipette and placed in a -80°C freezer for 5-10 minutes followed by thawing. This resultant fluid was then recentrifuged to pellet any remaining platelet bodies. The supernatant was extracted and sent to the bedside for use. If blood was drawn the day prior to the procedure, the PL was placed in a -20°C freezer overnight and re-thawed prior to use.

Injection Technique

**Figure 1:**
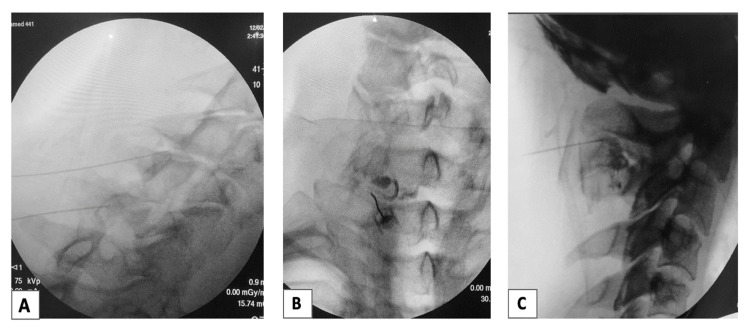
Companion case example fluoroscopy images. (A) Cervical facet injection, contralateral oblique view. (B) Cervical facet injection with epidural overflow, anteroposterior view. (C) Cervical interspinous ligament injection, lateral view.

Under sterile conditions, all patients received percutaneous injections under multiplanar fluoroscopic guidance. Patients received a combination of cervical facet joint injections with or without epidural overfill, cervical ligament injections, and/or transforaminal epidural injections (Figure 1). Specific levels for the injections and laterality were determined by symptoms, physical examination, and imaging studies. Patients either underwent cervical transforaminal injections or facet injections with fluid overfill to spread into the epidural space. Cervical facet capsules were also injected either with PRP, PL, or 12.5% hypertonic dextrose solution (prolotherapy). Cervical supraspinous and interspinous ligaments were also injected with PRP, PL, or prolotherapy when indicated.

Outcome measures

Neck pain treatment outcomes were measured by several patient-reported questionnaires, including the Numeric Pain Score (NPS), Functional Rating Index (FRI), and a modified Single Assessment Numeric Evaluation (SANE) [32-34]. The NPS is a scale ranging from 0 to 10, where 0 equates to no pain and 10 equates to the worst possible pain. The minimum clinically important difference (MCID) for the NPS is a decrease of 2 points. The FRI is on a scale of 1 to 100, where 0 indicates no functional limitations and 100 represents severe disability. The MCID for the FRI is a decrease of 8.4 points [35]. The modified-SANE asked patients to rate the percentage difference they felt post-treatment compared to pre-treatment. This is modified from the standard SANE rating because our question allowed answers from -100 (worsened) through +100 (improved); negative ratings were truncated as 0 to align with the standard SANE rating.

Statistical analysis

Continuous variables were described using the mean and standard deviation (SD). Individual differences between post-treatment time points and baseline (termed “change scores”) were calculated for NPS and FRI metrics, and the percentages of patients who met or exceeded the MCID for each metric were calculated. Wilcoxon signed-rank tests were used to assess whether post-treatment scores differed significantly from baseline. P-values below 0.05 were considered significant. All analyses were performed using R version 3.5.1 and RStudio version 1.1.456.

## Results

**Figure 2:**
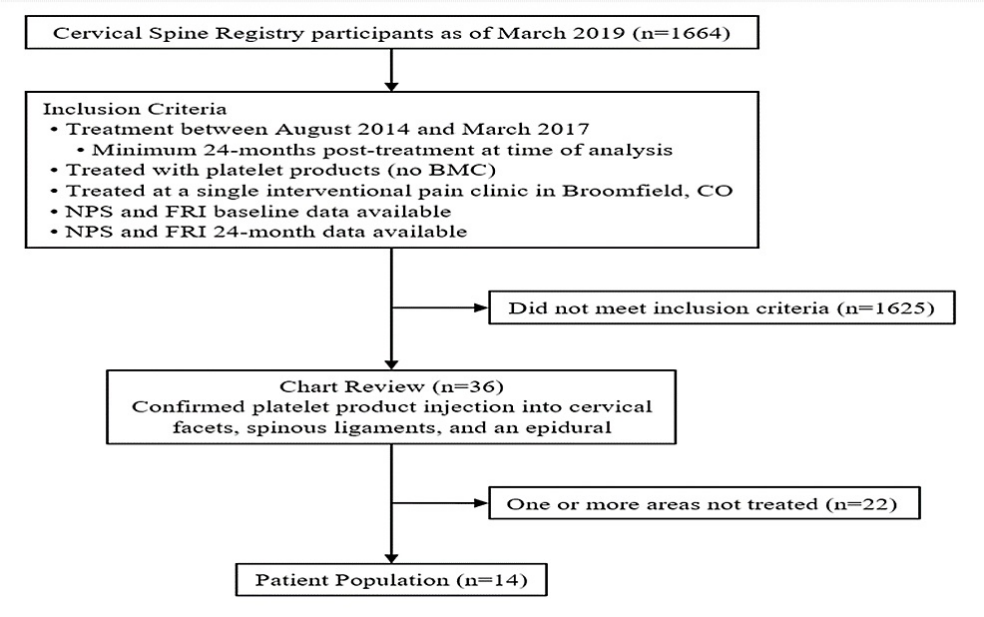
Flowchart of the included patients. BMC: bone marrow concentrate; NPS: Numeric Pain Score; FRI: Functional Rating Index

A total of 14 patients were isolated for the case series who met the inclusion criteria. See Figure 2 for a flowchart of the patient selection process. Patient age ranged from 49 to 81 years (mean: 59.8 years), of which 36% were male and 64% were female (Table 1). All patients underwent a combination of concentrated platelet injections into cervical facets, cervical interspinous, and supraspinous ligaments and epidural injections. Table 1 details patient diagnoses, structures targeted, and injectates used. The PRP used was leukocyte poor with a concentration range of 5-15x with a median of 8.5x. PL concentration ranged from 2-15x with a median of 2x. Two (14%) patients reported adverse events; one being a skin rash and increased post-procedure pain and the other itchiness at the site of injection. These were assessed by the treating physician and self-resolved.

**Table 1:** Patient details including demographics, diagnoses, injectates administered, additional treatment time-points, and adverse events. BMI: body mass index; DDD: degenerative disc disease; HNP: herniated nucleus pulposus; PRP: platelet-rich plasma; PLM: platelet lysate modified; PPP: platelet-poor plasma; M: male; F: female

Demographics				Diagnosis							Injectate						
Patient	Age	Gender	BMI	Instability	DDD	HNP	Facet arthropathy	Spondylosis	Radiculopathy	Stenosis	PRP	PLM	PPP	Prolotherapy	Epidural type	PRP booster	Adverse events
1	55	F	25.6	x	x				x			x		x	Overfill	23 months	
2	68	F	39.2	x			x					x			Overfill		Skin rash and pain
3	43	M	25.7	x	x		x		x	x	x	x			Overfill	4 months	
4	58	F	22.3		x	x	x	x				x	x		Traditional		
5	68	M	25.7	x			x			x	x	x			Overfill	7months, 11 months	
6	65	F		x			x				x	x			Overfill		
7	51	F	23.2	x	x		x	x	x			x		x	Traditional	6 weeks, 4 months	
8	53	M	24.4	x	x	x	x	x	x	x		x	x	x	Traditional	4 months	
9	60	M	25.1	x			x			x		x	x	x	Overfill		
10	81	M	23.2		x		x	x		x		x		x	Traditional		
11	49	F	29.0	x	x	x	x		x			x		x	Traditional	2 months	
12	57	F	24.3	x	x	x	x		x			x		x	Both	11 months	
13	69	F	23.0		x			x			x	x	x	x	Traditional		Itchiness at injection site
14	60	F	19.9	x			x					x	x		Overfill		

**Figure 3:**
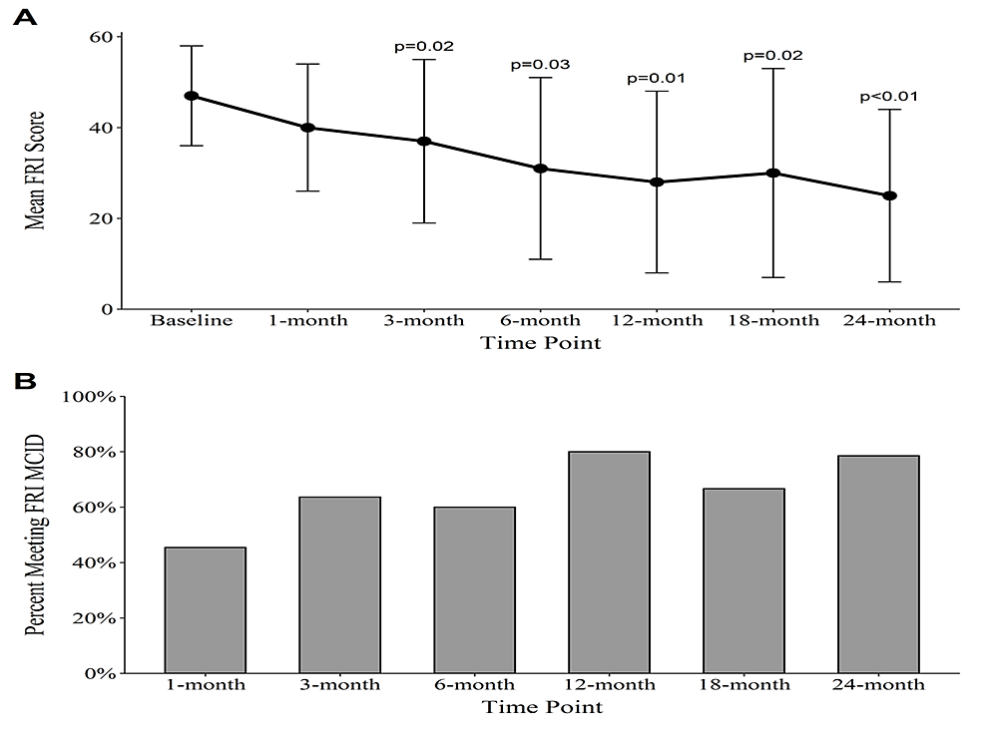
FRI outcomes. (A) Average FRI scores. P-values are representative of Wilcoxon signed-rank test comparisons between the time-point and baseline. N’s: 14, 11, 11, 10, 10, 12, 14. (B) Percentage of patients meeting the FRI MCID. FRI: Functional Rating Index; MCID: minimal clinically important difference

**Figure 4:**
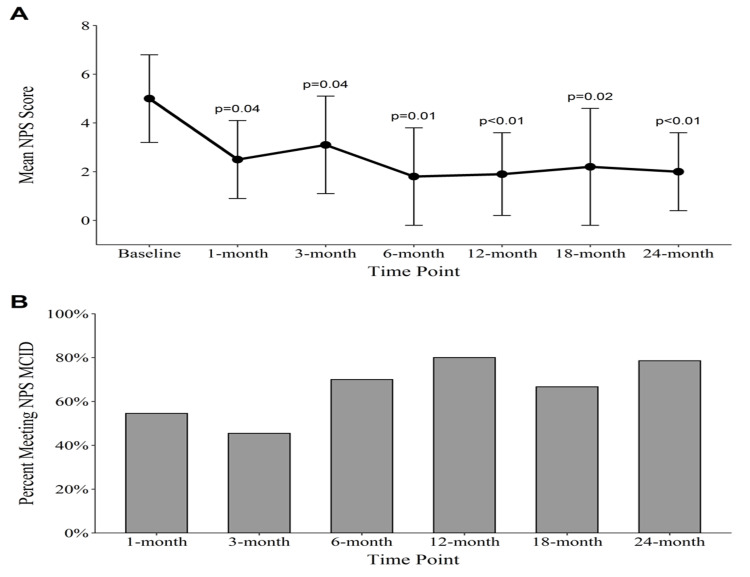
NPS outcomes. (A) Average NPS scores. P-values are representative of Wilcoxon signed-rank test comparisons between the time-point and baseline. N’s: 14, 11, 11, 10, 10, 12, 14; (B) Percentage of patients meeting the NPS MCID. NPS: Numeric Pain Score; MCID: minimal clinically important difference

**Figure 5:**
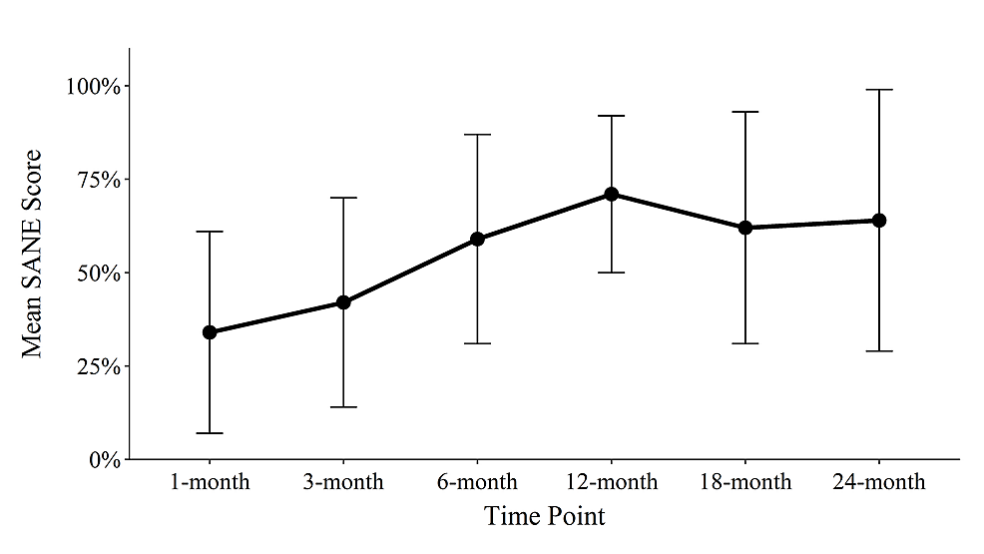
Average SANE scores per time-point. N’s: 11, 11, 10, 10, 12, 14. SANE: Single Assessment Numeric Evaluation

Raw outcome scores are shown in Table 2. Improvement in the mean FRI scores reached statistical significance at three, six, twelve, eighteen, and twenty-four months with an overall improvement of 27.3 (p = 0.004; confidence interval (CI) = [-33.8, -10.4]). The percentage of patients meeting the FRI MCID for function was lowest at one month (45%), highest at 12 months (80%), and was 79% at 24 months (Figure 3). The NPS change scores reached significance at all post-treatment time-points, with a mean decrease in NPS of 2.8 (p = 0.002; CI = [-4.5, -1.5]) at 24 months. The percentage of patients reaching the MCID for decreasing pain was lowest at 3 months (45%) and highest at 12 months (80%), with 79% of patients meeting or exceeding the MCID at 24 months (Figure 4). The modified SANE change scores showed an average reported percentage improvement of 64% at 24 months, with a peak average improvement of 71% at 12 months. Though the majority of patients had improvement in symptoms compared with prior to the procedure, two patients had no improvement in their symptoms at 24 months (Figure 5).

**Table 2:** Patient outcome data.

	Numeric Pain Scale							Functional Rating Index							Single Assessment Numeric Evaluation					
Patient	Baseline	1-month	3-month	6-month	12-month	18-month	24-month	Baseline	1-month	3-month	6-month	12-month	18-month	24-month	1-month	3-month	6-month	12-month	18-month	24-month
1	7		8	6	5	7	5	55.0		62.5	70.0	62.5	65.0	57.5		40	30	30	50	50
2	5			0	3	4	3	58.3			12.5	27.5	52.5	27.5			80	80	10	50
3	5	4	4			0	0	55.0	30.0	42.5			0.0	7.5	40	50			100	100
4	3	2	3		2	1	2	37.5	22.5	22.5		20.0	20.0	17.5	20	30		60	70	80
5	3	5	3	2	4	5	4	45.0	55.0	45.0	25.0	55.0	60.0	55.0	20	0	70	50	40	0
6	3	4	4	4		5	4	50.0	50.0	55.0	52.5		55.0	57.5	0	0	0		0	0
7	5	0	4				2	57.5	60.0	45.0				22.5	10	30				70
8	3	3	0	0	0	0	0	32.5	30.0	10.0	2.5	0.0	0.0	0.0	50	90	90	100	70	100
9	7	2	2	0	0	0	0	44.4	25.0	15.0	17.5	5.0	20.0	17.5	30	50	50	80	80	90
10	8	3	2	2	1	1	1	50.0	52.5	47.5	45.0	37.5	17.5	25.0	30	40	60	80	90	90
11	5	3	2	3	2	2	2	67.5	55.0	47.5	35.0	30.0	40.0	20.0	10	50	50	80	75	90
12	4	1	2	1	2	1	1	30.0	30.0	15.0	17.5	15.0	7.5	5.0	70	80	80	60	80	85
13	4	0		0	0	1	1	40.0	27.5		32.5	22.5	25.0	12.5	90		80	90	80	20
14	8						3	40.0						25.0						65

## Discussion

As has been previously described in the literature, the etiology of neck pain is multifactorial with a constellation of structural, chemical, and neurological factors likely to blame [7,19,36-38]. The burden of degenerative spine disorders contributes to decreased quality of life, higher likelihood of opiate utilization, decreased activity with the resultant indirect ill-effects on health, and significant healthcare expenditures [1,39,40]. Traditional interventional pain management techniques have necessitated a narrow treatment paradigm to a “pain generator” model used to target a limited number of structures as a temporizing measure rather than a disease-modifying, structural approach [12,13]. When considered through an FSU model, we see that pain is often preceded by varying degrees of segmental instability related to ligamentous laxity and/or degenerative disc height loss, predisposing to injury over time secondary to increased stress and inflammation in related structures [41,42]. Prolotherapy was the original regenerative medicine injection technique, with the underlying principle of efficacy encompassing the treatment of ligamentous instability [43-47]. The advent of PRP and its demonstrated efficacy in the treatment of injured tendons and ligaments, as well as osteoarthritis, offers a potentially more robust and comprehensive approach to an FSU treatment protocol [25,48-51].

The case series described here supports the use of autologous platelet-based injections performed under image guidance for the safe and effective treatment of axial neck pain with or without radiculopathy. Because the FSU treatment paradigm was used, the number of and specific levels treated varied based on patient-specific pathology. The injection sites were determined by clinical history, physical examination, and MRI studies. The case series demonstrates clinically significant improvements in pain and functional outcomes in patients undergoing cervical spine injections with concentrated platelets at the 24-month post-injection follow-up. When looking individually at the included patients, 12 patients improved in each category. Though the aggregate data show an overall significant improvement, two patients reported experiencing increased pain and a decrease in function on the FRI at 24 months. There are no clear factors unique to the two patients with poor outcomes and further research is needed to better clarify the predictors of positive responders. Regarding safety, two patients reported minor, self-resolving adverse events.

There are several limitations to this study. First, the data were collected via a registry with a relatively small sample size and without a control group for comparison. In addition, only 14 of the many patients treated met the criteria of 24-month follow-up data. Lack of full two-year outcomes data could be due to various reasons, including those lost to follow-up, surgical intervention, steroid injection(s), registry withdrawal request, or a new injury that confounded treatment outcomes, such as an automobile accident. Second, though the principles of treatment remained consistent among patients, there was a lack of standardization of PRP and PL concentrations. Some patients also underwent more than one treatment. Furthermore, at the time of this writing, RITs are not reimbursed by insurance in the United States. Therefore, there is often an out-of-pocket cost and monetary investment by the patient that could in theory lead to biased outcomes and increased motivation to perceive improvement. Demographics are also relatively self-selecting for this same reason. Previous public health analyses in the United States have shown a positive correlation between increasing wealth and better health [52]. The majority of the patients in the study, due to current restrictions in insurance coverage, are in a financial position to pay out-of-pocket costs for these treatments, and therefore may be predisposed to having an overall higher likelihood of good health due to other factors. Therefore, these results may not necessarily be generalizable to the entire population. However, the overall positive outcomes of this limited dataset do suggest the efficacy of such treatments and further provide a foundation from which larger, randomized controlled trials can be formulated.

## Conclusions

Our cases series compliments early data demonstrating clinical efficacy with injecting multiple components of the FSU with RITs in the treatment of painful spine disorders. PRP is a safe injectate that shows promise for effective treatment of axial neck pain when utilized in a thoughtful manner targeting ligamentous laxity, intraarticular facet arthritis, and nerve root irritation. Though this early data is encouraging, more comprehensive, randomized controlled trials including a larger number of patients are needed to further validate these findings. Given the significant impact of neck pain on quality of life for an aging population, an overreliance on opioid medications for the management of chronic musculoskeletal pain by providers, and the significant societal costs, both directly and indirectly, a more comprehensive treatment approach from a biomechanical perspective that offers the possibility of disease modification rather than symptom management is needed. This study further underscores the importance of continued research in this area to prove efficacy and help shape medical policy such that more patients have access to care that may prove to be, in the end, safer and more effective than that offered in our current treatment model.

## References

[1] Global Burden of Disease Study 2013 Collaborators (2015). Global, regional, and national incidence, prevalence, and years lived with disability for 301 acute and chronic diseases and injuries in 188 countries, 1990-2013: a systematic analysis for the Global Burden of Disease Study 2013. Lancet.

[2] Genebra CV, Maciel NM, Bento TP, Simeão SF, Vitta A (2017). Prevalence and factors associated with neck pain: a population-based study. Braz J Phys Ther.

[3] Strine TW, Hootman JM (2007). US national prevalence and correlates of low back and neck pain among adults. Arthritis Rheum.

[4] Hoy DG, Protani M, De R, Buchbinder R (2010). The epidemiology of neck pain. Best Pract Res Clin Rheumatol.

[5] Demyttenaere K, Bruffaerts R, Lee S (2007). Mental disorders among persons with chronic back or neck pain: results from the World Mental Health Surveys. Pain.

[6] Carey TS, Freburger JK, Holmes GM, Jackman A, Knauer S, Wallace A, Darter J (2010). Race, care seeking, and utilization for chronic back and neck pain: population perspectives. J Pain.

[7] Teichtahl AJ, McColl G (2013). An approach to neck pain for the family physician. Aust Fam Physician.

[8] Förster M, Mahn F, Gockel U, Brosz M, Freynhagen R, Tölle TR, Baron R (2013). Axial low back pain: one painful area--many perceptions and mechanisms. PLoS One.

[9] Manchikanti L, Pampati V, Falco FJ, Hirsch JA (2015). An updated assessment of utilization of interventional pain management techniques in the Medicare population: 2000 - 2013. Pain Physician.

[10] Manchikanti L, Pampati V, Kaye AD, Hirsch JA (2017). Cost utility analysis of cervical therapeutic medial branch blocks in managing chronic neck pain. Int J Med Sci.

[11] Manchikanti L, Falco FJ, Pampati V, Cash KA, Benyamin RM, Hirsch JA (2013). Cost utility analysis of caudal epidural injections in the treatment of lumbar disc herniation, axial or discogenic low back pain, central spinal stenosis, and post lumbar surgery syndrome. Pain Physician.

[12] Manchikanti L, Hirsch JA, Falco FJ, Boswell MV (2016). Management of lumbar zygapophysial (facet) joint pain. World J Orthop.

[13] Falco FJ, Manchikanti L, Datta S (2012). Systematic review of the therapeutic effectiveness of cervical facet joint interventions: an update. Pain Physician.

[14] Beitzel K, McCarthy MB, Cote MP (2013). The effect of ketorolac tromethamine, methylprednisolone, and platelet-rich plasma on human chondrocyte and tenocyte viability. Arthroscopy.

[15] Dragoo JL, Danial CM, Braun HJ, Pouliot MA, Kim HJ (2012). The chondrotoxicity of single-dose corticosteroids. Knee Surg Sports Traumatol Arthrosc.

[16] Sherman SL, Khazai RS, James CH, Stoker AM, Flood DL, Cook JL (2015). In vitro toxicity of local anesthetics and corticosteroids on chondrocyte and synoviocyte viability and metabolism. Cartilage.

[17] Zhang J, Tsuzuki N, Hirabayashi S, Saiki K, Fujita K (2003). Surgical anatomy of the nerves and muscles in the posterior cervical spine: a guide for avoiding inadvertent nerve injuries during the posterior approach. Spine (Phila Pa 1976).

[18] Carragee EJ, Hurwitz EL, Cheng I (2008). Treatment of neck pain: injections and surgical interventions: results of the Bone and Joint Decade 2000-2010 Task Force on Neck Pain and Its Associated Disorders. Eur Spine J.

[19] Aufiero D, Vincent H, Sampson S, Bodor M (2015). Regenerative injection treatment in the spine: review and case series with platelet rich plasma. J Stem Cells Res Rev Rep.

[20] Dumais R, Benoit C, Dumais A (2012). Effect of regenerative injection therapy on function and pain in patients with knee osteoarthritis: a randomized crossover study. Pain Med.

[21] Salamanna F, Veronesi F, Maglio M, Della Bella E, Sartori M, Fini M (2015). New and emerging strategies in platelet-rich plasma application in musculoskeletal regenerative procedures: general overview on still open questions and outlook. Biomed Res Int.

[22] Al-Ajlouni J, Awidi A, Samara O (2015). Safety and efficacy of autologous intra-articular platelet lysates in early and intermediate knee osteoarthrosis in humans: a prospective open-label study. Clin J Sport Med.

[23] Centeno C, Markle J, Dodson E, Stemper I, Hyzy M, Williams C, Freeman M (2017). The use of lumbar epidural injection of platelet lysate for treatment of radicular pain. J Exp Orthop.

[24] Taylor DW, Petrera M, Hendry M, Theodoropoulos JS (2011). A systematic review of the use of platelet-rich plasma in sports medicine as a new treatment for tendon and ligament injuries. Clin J Sport Med.

[25] Brown MN, Shiple BJ, Scarpone M (2016). Regenerative approaches to tendon and ligament conditions. Phys Med Rehabil Clin N Am.

[26] Campbell KA, Saltzman BM, Mascarenhas R, Khair MM, Verma NN, Bach BR Jr, Cole BJ (2015). Does intra-articular platelet-rich plasma injection provide clinically superior outcomes compared with other therapies in the treatment of knee osteoarthritis? A systematic review of overlapping meta-analyses. Arthroscopy.

[27] Mascarenhas R, Saltzman BM, Fortier LA, Cole BJ (2015). Role of platelet-rich plasma in articular cartilage injury and disease. J Knee Surg.

[28] Liou JJ, Rothrauff BB, Alexander PG, Tuan RS (2018). Effect of platelet-rich plasma on chondrogenic differentiation of adipose- and bone marrow-derived mesenchymal stem cells. Tissue Eng Part A.

[29] Smyth NA, Murawski CD, Fortier LA, Cole BJ, Kennedy JG (2013). Platelet-rich plasma in the pathologic processes of cartilage: review of basic science evidence. Arthroscopy.

[30] Miele VJ, Panjabi MM, Benzel EC (2012). Anatomy and biomechanics of the spinal column and cord. Handb Clin Neurol.

[31] Centeno CJ, Pitts J, Al-Sayegh H, Freeman MD (2015). Anterior cruciate ligament tears treated with percutaneous injection of autologous bone marrow nucleated cells: a case series. J Pain Res.

[32] Katz J, Melzack R (1999). Measurement of pain. Surg Clin North Am.

[33] Feise RJ, Michael Menke J (2001). Functional rating index: a new valid and reliable instrument to measure the magnitude of clinical change in spinal conditions. Spine (Phila Pa 1976).

[34] Shelbourne KD, Barnes AF, Gray T (2012). Correlation of a single assessment numeric evaluation (SANE) rating with modified Cincinnati knee rating system and IKDC subjective total scores for patients after ACL reconstruction or knee arthroscopy. Am J Sports Med.

[35] Childs JD, Piva SR (2005). Psychometric properties of the functional rating index in patients with low back pain. Eur Spine J.

[36] Manchikanti L, Manchikanti KN, Cash KA, Singh V, Giordano J (2008). Age-related prevalence of facet-joint involvement in chronic neck and low back pain. Pain Physician.

[37] Manchikanti L, Boswell MV, Singh V, Pampati V, Damron KS, Beyer CD (2004). Prevalence of facet joint pain in chronic spinal pain of cervical, thoracic, and lumbar regions. BMC Musculoskelet Disord.

[38] Falla DL, Jull GA, Hodges PW (2004). Patients with neck pain demonstrate reduced electromyographic activity of the deep cervical flexor muscles during performance of the craniocervical flexion test. Spine (Phila Pa 1976).

[39] Murray CJ, Atkinson C, Bhalla K (2013). The state of US health, 1990-2010: burden of diseases, injuries, and risk factors. JAMA.

[40] Davis MA, Onega T, Weeks WB, Lurie JD (2012). Where the United States spends its spine dollars: expenditures on different ambulatory services for the management of back and neck conditions. Spine (Phila Pa 1976).

[41] Linetsky FS, Miguel R, Torres F (2004). Treatment of cervicothoracic pain and cervicogenic headaches with regenerative injection therapy. Curr Pain Headache Rep.

[42] Hooper RA, Frizzell JB, Faris P (2007). Case series on chronic whiplash related neck pain treated with intraarticular zygapophysial joint regeneration injection therapy. Pain Physician.

[43] Jensen KT, Rabago DP, Best TM, Patterson JJ, Vanderby R Jr (2008). Early inflammatory response of knee ligaments to prolotherapy in a rat model. J Orthop Res.

[44] Centeno CJ, Elliott J, Elkins WL, Freeman M (2005). Fluoroscopically guided cervical prolotherapy for instability with blinded pre and post radiographic reading. Pain Physician.

[45] Hackett GS (1958). Ligament and tendon relaxation treated by prolotherapy. http://www.prolotherapyflorida.com/Portals/0/Ligament%20And%20Tendon%20Relaxation,%20Third%20Edition.pdf.

[46] Reeves KD, Hassanein K (2000). Randomized, prospective, placebo-controlled double-blind study of dextrose prolotherapy for osteoarthritic thumb and finger (DIP, PIP, and trapeziometacarpal) joints: evidence of clinical efficacy. J Altern Complement Med.

[47] Rabago D, Best TM, Beamsley M, Patterson J (2005). A systematic review of prolotherapy for chronic musculoskeletal pain. Clin J Sport Med.

[48] Yuan T, Zhang CQ, Wang JH (2013). Augmenting tendon and ligament repair with platelet-rich plasma (PRP). Muscles Ligaments Tendons J.

[49] Marmotti A, Rossi R, Castoldi F, Roveda E, Michielon G, Peretti GM (2015). PRP and articular cartilage: a clinical update. Biomed Res Int.

[50] Laver L, Marom N, Dnyanesh L, Mei-Dan O, Espregueira-Mendes J, Gobbi A (2017). PRP for degenerative cartilage disease: a systematic review of clinical studies. Cartilage.

[51] Wu J, Zhou J, Liu C (2017). A prospective study comparing platelet-rich plasma and local anesthetic (LA)/corticosteroid in intra-articular injection for the treatment of lumbar facet joint syndrome. Pain Pract.

[52] Braveman PA, Cubbin C, Egerter S, Williams DR, Pamuk E (2010). Socioeconomic disparities in health in the United States: what the patterns tell us. Am J Public Health.

